# Enhancing artificial intelligence chatbot dependability in Parkinson's disease patients: future of care

**DOI:** 10.1590/1980-5764-DN-2025-0368

**Published:** 2026-04-03

**Authors:** Akhilesh Vikram Singh, Anudwipa Singh

**Affiliations:** 1 Graphic Era Deemed to be a University, Department of Biotechnology, Dehradun, Uttarakhand, India.; 2 Sri Sai Jyothi College of Pharmacy, Hyderabad, Telangana, India.

Dear Editor,

Parkinson's disease (PD) is a common neurodegenerative disorder characterized by motor and non-motor symptoms such as tremors, bradykinesia, and dystonias, which lead to difficulty in speaking^1^. The prevalence of PD rises with increasing age, with more than 10 million people affected worldwide^2^. Genetic and environmental factors, such as pollution and pesticides, contribute to the increased risk of PD. There is no cure for PD to date, and management lies in therapies such as medicines, rehabilitation, and surgery to limit the progression of the disease^3^.

Artificial intelligence (AI) has emerged as a solution for PD patients, as technologies can delay or improve PD symptoms^4^. Various AI applications have been developed for the management of PD, such as deep-learning-based pose estimation algorithms (Convolution Pose Machines) to detect disease severity or levodopa-induced dyskinesia, clinical decision support systems, Microsoft Kinect multimodal off-the-shelf sensors to assess adherence to drug treatment, epiNet, a novel artificial gene regulatory network to monitor movement, robotic systems and AI in gait management, and home-based telemedicine systems for diagnosis and management of PD^5-7^. These technologies help improve the quality of life of patients with PD and reduce the financial burden on them.

AI chatbots have helped to get a better understanding of patient responses in PD. AI chatbots are technologies that utilize automatic speech recognition and natural language processing to simulate communication with users. The use of AI chatbots in healthcare has been increasing in recent years^8^. The 24-h availability of chatbots can reduce the burden of healthcare workers involved in the management of PD patients by collecting patients’ health information daily and facilitating patient communication^9^. AI chatbots have been found to enhance smiles and speech in PD patients. Smile and speech features, in turn, may help to detect the motor, cognitive, and mental status of PD patients^10^.

Available data on AI chatbots highlights their potential as a promising solution for managing PD. AI chatbots can assess adherence to drug treatment and be incorporated into telemedicine systems for improved diagnosis and management of PD. AI-assisted apps incorporating chatbots can be developed to communicate with PD patients. There is a lack of research on the use of AI chatbots in treating PD, suggesting the need for future research in this area.

## Data Availability

The datasets generated and/or analyzed during the current study are available from the corresponding author upon reasonable request.
